# The Efficacy and Safety of Intranasal Formulations of Ketamine and Esketamine for the Treatment of Major Depressive Disorder: A Systematic Review

**DOI:** 10.3390/pharmaceutics15122773

**Published:** 2023-12-13

**Authors:** Ludivine Boudieu, Myriam Mennetrier, Pierre-Michel Llorca, Ludovic Samalin

**Affiliations:** Department of Psychiatry, CHU Clermont-Ferrand, CNRS, Clermont Auvergne INP, Institut Pascal (UMR 6602), University of Clermont Auvergne, 63000 Clermont-Ferrand, France; lboudieu@chu-clermontferrand.fr (L.B.); mmennetrier@chu-clermontferrand.fr (M.M.); pmllorca@chu-clermontferrand.fr (P.-M.L.)

**Keywords:** ketamine, esketamine, intranasal formulation, major depressive disorder, treatment-resistant depression

## Abstract

Ketamine and its enantiomers represent an innovative glutamatergic agent as a treatment for individuals with treatment-resistant depression (TRD) and major depressive disorder (MDD) with suicidal ideation and behavior. Intranasal (IN) formulations could allow for quick onset of action on depressive symptoms as well as a reduction in side effects by bypassing the blood–brain barrier compared with administration via the intravenous route. The aim of this review was to provide an up-to-date analysis of the data on the efficacy and safety of IN ketamine and IN esketamine for the treatment of MDD. A systematic review following PRISMA guidelines was conducted. Databases (PubMed, Embase, MEDLINE, PsycINFO, and Google Scholar) were searched to capture articles about IN ketamine or IN esketamine for MDD. This systematic review highlighted the interest in IN routes of ketamine and esketamine for MDD patients with TRD or active suicidal ideation. They provide a rapid onset of antidepressant action within the first hours after administration. Nevertheless, the evidence of efficacy is stronger for IN esketamine than for IN ketamine in MDD patients. The safety profile appears to be acceptable for IN esketamine but requires further studies, and a more accurate IN delivery device is required for ketamine.

## 1. Introduction

In 2019, the World Health Organization (WHO) estimated that 280 million people lived with major depressive disorder (MDD) (i.e., 3.8% of the global population) [1]. This mental disorder is characterized by the presence of a depressed mood and anhedonia accompanied by four or more other symptoms of depression for at least 2 weeks (according to the Diagnostic and Statistical Manual of Mental Disorders, Fifth edition, DSM-5). MDD is responsible for loss of quality of life and impairment in daily functioning and is associated with high mortality (10 to 20% of patients with MDD attempt suicide over their lifetime) and years of life lost (10-year reduction in life expectancy) [2,3].

Despite a large antidepressant therapeutic arsenal, only one-third of patients achieve remission after the first treatment line, and one-third of patients develop treatment-resistant depression (TRD) [4,5]. TRD is defined as a reduction of less than 25% in MDD symptom severity after two or more prior treatment trials with different antidepressants (ADs), despite adequate dosage and duration for the current episode [6].

Recommendations for the treatment of these patients are based on the following [7]:

- Pharmacological strategies: switching strategies (concurrent switch, overlapping switch, or sequential switch) or combination strategy, i.e., adding another AD or an add-on strategy (with lithium or quetiapine, for example) to ongoing AD;
- Psychotherapy approaches (supportive therapy and psychoeducational intervention) can be used too, but only in combination with an AD;
- Brain stimulation techniques with a preferential choice for electroconvulsive therapy (ECT) and repetitive transcranial magnetic stimulation (rTMS) in monotherapy or in combination with the current AD.

Unfortunately, conventional ADs have a time-to-onset effect of 3 to 7 weeks, during which time patients remain symptomatic, with an increase in biological and social impact and risk of self-harm [5]. Furthermore, the accessibility of these brain stimulation techniques is limited for various reasons, such as eligibility (need for anesthesia) or patient acceptability.

Despite the large variety of treatment options currently available for the management of TRD, its annual incidence and prevalence remain high, going up to 25.8 per 10,000 persons and 37.6 per 10,000 persons, respectively (data from French health insurance, dating from 2020) [8]. These patients have an increased all-cause mortality of 35% compared to non-TRD MDD patients [9]. Even when MDD patients with TRD respond to treatment, the overall rate of relapse while continuing treatment with the same AD increases with the number of failures (two failed trials: 65% of relapse within 3.1 months; three failed trials: 71.1% of relapse within 3.3 months) [5]. Moreover, TRD episodes last longer and generate more direct and indirect costs than non-TRD MDD [10,11].

Scientific research on depression pathophysiology has highlighted the implication of glutamatergic transmission, with histopathological changes in neural circuits that modulate emotional behavior [12]. This evidence led to the investigation of glutamatergic agents such as ketamine that act on this glutamatergic transmission. Ketamine, introduced into clinical practice in the 1960s, is an anesthetic agent with unique pharmacological properties. Ketamine’s pharmacological effects range from the induction and maintenance of anesthesia to analgesia and sedation, depending on the dose [13]. Ketamine has low cardiorespiratory depressant and sympathomimetic effects and can induce a dissociative state [14]. In clinical practice, ketamine is used for anesthesia, acute and chronic pain control, and, more recently, as a potential antidepressant.

Ketamine is a non-competitive N-methyl-D-Aspartate (NMDA) receptor antagonist that acts on the glutamatergic system. This antagonism leads to increased glutamate release, which, in turn, leads to increased AMPA receptor stimulation and neurotrophic signalization acting positively on synaptic function (notably in brain regions involved in mood regulation and emotional behavior). The effects of ketamine are also probably due to its action on the dopaminergic system (particularly via the D_2_ receptor) by restoring dopaminergic neurotransmission in brain regions that control motivation and reward and reducing stimulation in the regions involved in anhedonia. Ketamine also acts on other types of receptors, such as serotonin receptors 5–HT_2_ and 5-HT_1B_, opioids receptors µ and κ, and muscarinic receptor M_1_ [15,16]. Thus, intravenously administered ketamine at a subanesthetic dose (0.5 mg/kg) demonstrated a robust and rapid improvement in depressive symptoms in MDD patients with TRD [17,18,19]. Nevertheless, the need for intravenous administration may reduce treatment acceptability for outpatients and is not suitable for long-term treatment.

Given the limitations of a treatment requiring intravenous administration and the low bioavailability of this drug via the oral route (17 to 27%) [20], ketamine administered intranasally could be a safer and more convenient alternative for patients while maintaining good bioavailability of around 45% [13]. Intranasal (IN) formulations also could allow for quick onset of action on depressive symptoms and a reduction in the drug dose and some systemic side effects by bypassing the blood–brain barrier in comparison with intravenous administration [21,22].

In 2014, the first study showing the antidepressant effect of a single dose (50 mg) of ketamine via an IN route in MDD patients who had failed to respond to at least one prior antidepressant trial was published [23].

The chemical structure of ketamine consists of a racemic mixture of two enantiomers (mirror image molecules): (*S*+)-ketamine (esketamine) and (*R*−)-ketamine (arketamine). These enantiomers have similar pharmacokinetic (PK) properties but different pharmacodynamic characteristics. Esketamine has a better affinity and four times more potent activity than the (*R*)-enantiomer for NMDA receptors (the inhibition constant Ki of esketamine for the NMDA receptor is 0.30 µM compared with 1.40 µM for arketamine) [16,24].

The different affinities of the enantiomers correlate with the different antidepressant profiles of these compounds in animal models of depression. Preclinical data suggest that arketamine has longer-lasting therapeutic effects and better safety than esketamine [25]. However, the only randomized placebo-controlled trial evaluating the antidepressant effects of intravenous arketamine did not suggest the efficacy of arketamine as compared to a placebo in TRD subjects [26]. Only one open-label pilot study suggested that arketamine might produce antidepressant effects in seven TRD patients, similar to previous reports on animal models [27]. Pending a higher level of evidence for arketamine in TRD, a comparison between ketamine (a racemic mixture of arketamine and esketamine) and esketamine could be of interest, especially if ketamine were administered via the same intranasal route.

An IN device has been developed in a single-use, ready-to-use form that delivers a total of 28 mg of esketamine in two sprays (one spray per nostril). The bioavailability of esketamine via IN administration is equivalent to that of IN ketamine and is around 50% when administering 56 or 84 mg [28].

IN esketamine administration in combination with conventional antidepressants SSRIs (selective serotonin reuptake inhibitors) or SNRIs (serotonin-norepinephrine reuptake inhibitors) was approved in 2019 by the U.S. Food and Drug Administration (FDA) and European Medicines Agency (EMA) for MDD patients with TRD or for the rapid reduction of depressive symptoms in MDD patients, which, according to clinical judgment, constitute a psychiatric emergency (i.e., acute suicidal ideation or behavior) [29].

The aim of this review was to provide an up-to-date analysis of the data on the efficacy and safety of intranasal formulations of ketamine and its enantiomers for the treatment of MDD.

## 2. Materials and Methods

### 2.1. Methods

This review was conducted in accordance with Preferred Reporting Items for Systematic Reviews and MetaAnalyses (PRISMA).

### 2.2. Literature Search

A search of the electronic databases PubMed, Embase, MEDLINE, PsycINFO, and Google Scholar was conducted to identify peer-reviewed articles in French and English published between December 2014 and October 2023. The time period selected was based on the first publication of a clinical study on IN ketamine in patients with MDD [23].

Search terms were selected to capture any articles about IN ketamine or IN esketamine and MDD including TRD: («depressive disorder, treatment-resistant» [MeSH Terms] OR «depressive disorder, major» [MeSH Terms] AND (resistance) OR (depressive disorder/drug therapy) [MeshTerms] AND («ketamine») OR («esketamine») AND («administration, intranasal» [MeSH Terms] OR «intranasal» [Text Word] OR «nasal sprays» [MeSHTerms].

### 2.3. Study Selection Process

Articles were included if they met the below criteria:

- They included patients with an MDD diagnosis;
- They included participants who were treated with IN ketamine or IN esketamine as an add-on or not;
- They were randomized controlled trials (RCTs) or open-label or observational studies;
- They measured the efficacy and safety of ketamine or esketamine treatment.

Articles were excluded if they met the below criteria:

- They were reviews, post hoc studies, case reports, qualitative studies, or protocols;
- They were animal studies;
- The treatment was not administered via an IN formulation;
- They included participants who did not meet a diagnosis of MDD (i.e., bipolar disorder diagnosis).

### 2.4. Data Extraction

We used a data extraction template developed for this systematic review for the extraction of the following characteristics and data:

- Article reference details, author, and year of publication;
- Study characteristics: study design, primary outcomes, side effects, duration of study, main findings;
- Population studied characteristics: sample size, mean age, % of female participants, clinical state, Montgomery–Asberg Depression Rating Scale (MADRS) score;
- Treatment: ketamine, esketamine, dosage, add-on or not;
- Efficacy characteristics: MADRS score;
- Safety/tolerance characteristics: side effects, % of each side effect.

### 2.5. Methodological Quality Assessment

The quality of each included study was assessed by two independent authors (LB and LS) using the NIH study quality assessment tools (National Institute of Health 2021). The tools used were adapted to each study design (Quality Assessment of Controlled Interventions Studies, Quality Assessment Tool for Observational Cohort and Cross-Sectional Studies). In the case of discrepancies, the two raters had to discuss them amongst themselves until a consensus was reached.

## 3. Results

**Figure 1:**
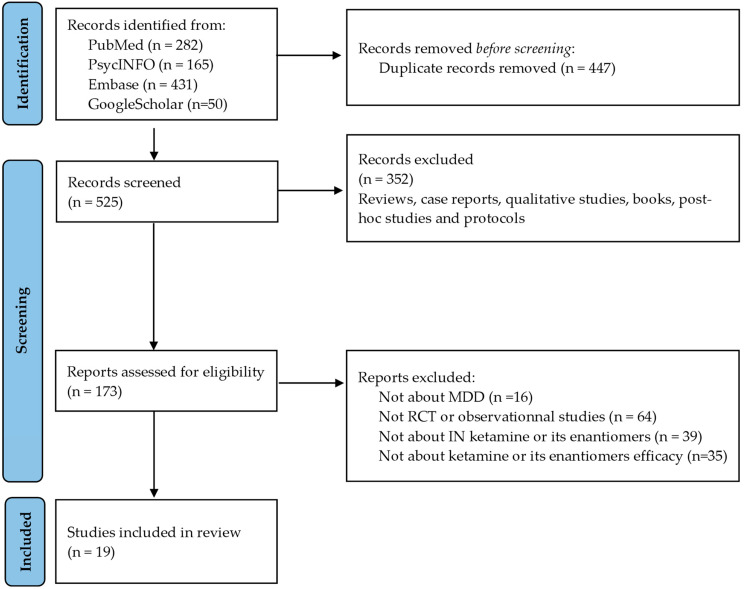
PRISMA flowchart.

The search retrieved 525 records after duplicates were removed. Among them, 173 abstracts were considered eligible and 352 were excluded. A full-text review of the 173 selected articles was conducted. A total of nineteen studies were included in the review (Table 1 and Table 2): twelve randomized placebo-controlled trials [23,30,31,32,33,34,35,36,37,38,39,40], one randomized active-controlled trial [41], one open-label study [42], and five observational studies [43,44,45,46,47]. Most eligible abstracts were excluded due to the study’s design (not randomized, controlled trials (RCTs) or open-label or observational studies). Figure 1 is a flowchart of the considered and ultimately selected studies, following the PRISMA statements.

### 3.1. Characteristics of Studies and Participants

Except for two RCTs of IN ketamine [23,40], all other studies evaluated the efficacy and safety of IN esketamine.

A total of 4860 participants were included in these nineteen studies, including 3001 women (61.7%) and 1859 males (38.3%). In eighteen out of nineteen studies, participants were middle-aged adults (18 to 65 years old). One study selected only participants aged 65 or more [34].

Ten RCTs [32,34,36,37,38,39,41], one open-label study [42], and the four observational studies [43,46,47] included MDD patients with TRD or with inadequate response to their ongoing AD, and three RCTs included MDD patients with active suicidal ideation [31,33,35].

The definition of TRD varied among the selected studies. Reif et al. (2023), Takahashi et al. (2021), and Daly et al. (2019) defined TRD as the absence of response to ≥1 but <5 different oral ADs (adequate dosage and for an adequate duration) [32,36,41]. For Chen et al. (2023), Estrade et al. (2023), Singh et al. (2023), Ochs-Ross et al. (2020), Fedgchin et al. (2019), Popova et al. (2019), Wajs et al. (2020), Brendle et al. (2022), Martinotti et al. (2022), Samalin et al. (2022), and Galves et al. (2018), TRD was defined as no response to an adequate trial (dose, duration, and adherence) after two or more ADs [30,34,37,38,40,42,43,44,45,46,47]. Daly et al. (2018) described TRD as a history of inadequate response to two or more ADs with at least one inadequate response in the current depressive episode [39].

For the three RCTs, including MDD patients with active suicidal ideation, recruitment was conducted in emergency departments, and patients were then transferred to psychiatric units [31,33,35].

**Table 1:** Characteristics of randomized controlled studies and open-label studies included in the systematic review.

Author, Year	Study Design	Population Study	Primary Outcome	Duration	Study Drugs	Main Findings
MDD patients with TRD						
Chen et al., 2023 [30]	Placebo-controlled RCT	250 MDD patients with TRD	Change in MADRS total score from baseline to week 4	4 weeks	IN ESK 56–84 mg vs. placebo	No statistical difference in MADRS score was observed between ESK and placebo groups from baseline to week 4.
Reif et al., 2023 [41]	Active-controlled RCT (ESCAPE-TRD)	676 MDD patients with TRD	Remission rate at week 8	32 weeks	IN ESK 28–84 mg vs. quetiapine	A significantly greater remission rate was observed in the ESK group compared to the quetiapine group at week 8 (27.1% vs. 17.6%, *p* = 0.003, respectively).
Takahashi et al., 2021 [32]	Placebo-controlled RCT	202 MDD patients with TRD	Change in MADRS score from baseline to week 4	4 weeks (DB phase) 24 weeks (OL phase)	IN ESK 28, 56, and 84 mg vs. placebo	No statistical difference in MADRS score was observed between ESK and placebo groups from baseline to week 4.
Ochs Ross et al., 2020 [34]	Placebo-controlled RCT (TRANS FORM-3)	138 MDD patients with TRD and ≥65 years old	Change in MADRS score from baseline to week 4	4 weeks	IN ESK 28–84 mg vs. placebo	No statistical difference in MADRS score was observed between ESK and placebo groups from baseline to week 4.
Wajs et al., 2020 [42]	OL trial (SUSTAIN-2)	802 MDD patients with TRD	Change in MADRS score from baseline to week 4 (IND phase) and from week 5 to week 52 (OP/MAINT phase)	52 weeks	IN ESK 28–84 mg	MADRS score decreased in the IND phase (baseline to week 4) (mean [SD] change: −16.4 [8.76]) and persisted during the OP/MAINT phase (week 5 to week 52) (mean [SD] change: 0.3 [8.12]).
Daly et al., 2019 [36]	Placebo-controlled RCT	297 MDD patients with TRD	Time to relapse in patients with stable remission after IND phase (week 4)	Until relapse	IN ESK 56–84 mg vs. placebo	Significant delay to relapse was observed in the ESK group compared to the placebo group (HR, 0.49; 95% CI 0.29–0.84; *p* = 0.003).
Fedgchin et al., 2019 [37]	Placebo-controlled RCT (TRANS FORM-1)	342 MDD patients with TRD	Change in MADRS score from baseline to week 4	4 weeks	IN ESK 56 and 84 mg vs. placebo	No statistical difference in MADRS score was observed between ESK and placebo groups from baseline to week 4.
Popova et al., 2019 [38]	Placebo-controlled RCT (TRANS FORM-2)	227 MDD patients with TRD	Change in MADRS score from baseline to week 4	4 weeks	IN ESK 56–84 mg vs. placebo	Significantly greater improvement in MADRS score was observed in the ESK group compared to the placebo group from baseline to week 4 (LS mean difference [SE]: −4.0 [1.69], *p* = 0.02).
Daly et al., 2018 [39]	Placebo-controlled RCT	67 MDD patients with TRD	Change in MADRS score from baseline to week 1 and week 2	2 weeks (DB phase) d15–d74 (OL phase)	IN ESK 28, 56, and 84 mg vs. placebo	Significantly greater improvement in MADRS score was observed in ESK groups compared to the placebo group in both periods (mean difference from placebo [SE]: ESK 28 mg: −4.2 [2.09], *p* = 0.02; ESK 56 mg: −6.3 [2.07], *p* = 0.001; ESK 84 mg: −9.0 [2.13], *p* < 0.001).
Galves et al., 2018 [40]	Active-controlled RCT	5 MDD patients with TRD	Feasibility and safety of repeated IN ketamine	4 weeks	IN ketamine 100 mg vs. midazolam 4.5 mg	The study was stopped early due to poor tolerability after the inclusion of five patients.
Lapidus et al., 2014 [23]	Placebo-controlled RCT	20 MDD patients with TRD	Change in MADRS score from baseline to 24 h	7 days	IN ketamine 50 mg vs. placebo	Significantly greater improvement in MADRS score was observed in the ketamine group compared to the placebo group from baseline to 24 h (*t*-test = 4.39, *p* < 0.001).
MDD patients with active suicidal ideation						
Ionescu et al., 2021 [31]	Placebo-controlled RCT (ASPIRE II)	230 MDD patients with ASI	Change in MADRS score from baseline to 24 h	4 weeks	IN ESK 84 mg vs. placebo	Significantly greater improvement in MADRS score was observed in the ESK group compared to the placebo group from baseline to 24 h (LS mean difference [SE]: −3.9 [1.39]; −1.11; *p* = 0.006).
Fu et al., 2020 [33]	Placebo-controlled RCT (ASPIRE I)	226 MDD patients with ASI	Change in MADRS score from baseline to 24 h	4 weeks	IN ESK 84 mg vs. placebo	Significantly greater improvement in MADRS score was observed in the ESK group compared to the placebo group from baseline to 24 h (LS mean difference [SE]: −3.8 [1.39]; *p* = 0.006).
Canuso et al., 2019 [35]	Placebo-controlled RCT	68 MDD patients with imminent suicide risk	Change in MADRS score from baseline to 4 h	4 weeks	IN ESK 84 mg vs. placebo	Significantly greater improvement in MADRS score was observed in the ESK group compared to the placebo group from baseline to 4 h (LS mean difference [SE]: −5.3 [2.10], *p* = 0.015).

Abbreviations: ASI: active suicidal ideation; d: days; ESK: esketamine; HR: hazard ratio; IN: intranasal; IND phase: induction phase; LS: least-squares; MADRS: Montgomery–Asberg Depression Rating Scale; MDD: major depressive disorder; OL: open-label; OP/MAINT phase: optimization/maintenance phase; RCT: randomized controlled trial; SE: standard error; SD: standard deviation; TRD: treatment-resistant depression.

**Table 2:** Characteristics of observational studies included in the systematic review.

Author, Year	Study Design	Population Study	Main Outcomes	Duration	Study Drug	Main Findings
Estrade et al., 2023 [43]	OS	105 MDD patients with TRD	Define trajectories of ESK response using MADRS score	5 months	IN ESK 28–84 mg	After two ESK administrations, the MADRS score predicted the 90-day trajectories of response with an accuracy of 80%.
Singh et al., 2023 [44]	OS	62 MDD patients with TRD	Change in QIDS-SR score and time to achieve response and remission	Up to 6 weeks	Ketamine IV 0.5 mg/kg vs. IN ESK 56–84 mg	There was no significant difference in response and remission rates between IV and IN routes. A significantly faster time to remission was observed in the ketamine IV group compared to the IN ESK group (HR = 5.0, *p* = 0.02).
Brendle et al., 2022 [45]	OS	171 MDD patients	Change in GAD-7 and PHQ-9 scores	1 to 71 treatment sessions	IN ESK 56–84 mg	Significant reduction in PHQ-9 and GAD-7 scores was observed between baseline and last available treatment (PHQ-9 = mean [SD]: 16.7 [5.82] vs. 12 [6.38], *p* < 0.001; GAD-7 = mean [SD]: 12.0 [5.8] vs. 8.7 [5.62], *p* < 0.001).
Martinotti et al., 2022 [46]	OS	116 MDD patients with TRD	Change in MADRS score and response and remission rates	3 months	ESK 28–84 mg	Significant improvement in MADRS score was observed at months 1 (T1) and 3 (T2) (Student *t*-test baseline vs. T1: *t* −15.79, *p* < 0.0001; T2 vs. baseline: *t* 18.07, *p* < 0.0001).
Samalin et al., 2022, [47]	OS	66 MDD patients with TRD	Characteristics of TRD patients receiving IN ESK, change in MADRS score	30 days (median treatment exposure)	ESK 28–84 mg	A decrease in MADRS score by 36% was observed from baseline to week 4 (mean MADRS score: 30.9 vs. 19.5, respectively).

Abreviations: ESK: esketamine; GAD: general anxiety disorder; HR: hazard ratio; IN: intranasal, IV: intravenous; MADRS: Montgomery–Asberg Depression Rating Scale; MDD: major depressive disorder; OS: observational study; PHQ-9: patient health questionnaire; QIDS-SR: Quick Inventory of Depressive Symptomatology-Self Report; SD: standard deviation; TRD: treatment-resistant depression.

### 3.2. Characteristics of Interventions, Drugs, and Control Conditions

Depending on the studies, the patients were treated with their current oral AD (in monotherapy or in combination with an adjunctive agent) at the time of randomization [31,33,35] or a new oral AD was initiated (mainly SSRIs or SNRIs).

IN esketamine was administrated twice weekly during the induction phase (first 4 weeks) in all included studies (except for Daly et al., 2018, where IN esketamine was administrated twice weekly for 2 weeks [39]). Some studies imposed a fixed dose (only possibility to decrease the dose for intolerance or side effects) [31,32,37,39,40] while, for others studies, the choice of dose was based on the investigator’s clinical judgment.

After 4 weeks, IN esketamine administration frequency and/or dose were adjusted [32,36,39,41,42,43,45,46,47]. Frequency was decreased to once weekly and/or every other week during the optimization/maintenance phase. Doses were adjusted based on the investigator’s clinical judgment of the efficacy and tolerability.

Patients self-administered IN esketamine at the study site under the supervision of a healthcare professional or staff member [23,30,31,33,34,35,36,37,38,39,40,41,42,43,44,46,47]. Only two studies did not specify the method of administration of IN esketamine [32,45].

IN ketamine was administered at 10 mg per IN application for a total of 50 mg of the study drug (five IN applications separated by 5 min) or a total of 100 mg of the study drug (10 IN applications separated by 5 min) as a single dose [23,40]. In the first study, administrations were provided over 20 min by an anesthesiologist in a clinical research unit [23]. In the second study, patients self-administered IN ketamine under the supervision of a research staff member [40]. Research staff extensively trained them in self-administering IN sprays, practicing with saline-filled atomization devices on a separate day before the first treatment.

### 3.3. Efficacy of IN Ketamine and IN Esketamine

MADRS was used in all included studies to measure depressive symptom severity. In two studies, the Quick Inventory of Depressive Symptomatology-Self Report (QIDR-SR) was also used to evaluate depressive symptom severity from the patient’s point of view [23,44].

Suicidal ideation or behavior was also evaluated in three RCTs with scales such as the Suicide Ideation and Behavior Assessment Tool (Clinical Global Impression-Severity of Suicide-revised (CGI-SS-r), Clinician Global Impression-Imminent Suicide Risk (CGI-SR-I), and Frequency of Suicidal Thinking (FoST)) [31,33,35].

#### 3.3.1. Efficacy in MDD Patients with TRD

The short-term efficacy of IN ketamine and IN esketamine was evaluated by the MADRS score changes at different time points after the first administration in eight double-blind, randomized, placebo-controlled trials [23,30,32,34,37,38,39,40]. However, the randomized, double-blind, placebo-controlled pilot study from Galvez et al. (2018) compared a 4-week course of eight treatments of 100 mg IN ketamine vs. 4.5 mg midazolam was stopped early due to safety concerns [40].

One randomized placebo-controlled trial evaluated changes in MADRS scores 4 h after IN ketamine administration [23]. An early effect was found in this study, with a significantly greater improvement in MADRS score changes with IN ketamine compared to the placebo (−9.75, *p* < 0.05, respectively).

Two randomized placebo-controlled trials evaluated the efficacy of IN ketamine [23] and IN esketamine [32] 24 h after the first administration. Statistical analysis found a significant decrease in MADRS scores in the IN ketamine and IN esketamine groups in comparison with the placebo group.

Two randomized placebo-controlled trials evaluated the efficacy of IN ketamine [23] or IN esketamine [32] on day 7/8 after the first administration. Lapidus et al. (2014) found a significant improvement in depressive symptoms at day 7 in patients treated with IN ketamine in comparison to the placebo [23]. Daly et al. (2018) also showed a significant decrease in MADRS scores in the IN esketamine group compared to the placebo group on day 8 after the first administration [39].

Six randomized placebo-controlled trials evaluated IN esketamine efficacy at day 25/28 after the first administration [30,32,34,37,38,39]. Two of the six RCTs showed a significantly higher improvement in MADRS scores in the IN esketamine group than in the placebo group [38,39]. In the four other RCTs with the same endpoint analysis, no significant difference between IN esketamine and placebo groups was demonstrated [30,32,34,37]. Nevertheless, prespecified analysis by age at baseline in the OchsRoss et al. study (2020) showed a significant improvement in MADRS scores in the IN esketamine (28, 56, or 84 mg) groups compared to the placebo group for patients aged 65 to 74 years (−4.9, *p* = 0.017) [34]. No difference was observed in patients aged over 75 years.

The efficacy of IN esketamine was also evaluated in comparison with quetiapine augmentation in one randomized active-controlled trial, ESCAPE-TRD, in MDD patients with TRD receiving an ongoing SSRI/SNRI [41]. This study consisted of an 8-week acute phase (primary endpoint) followed by a 24-week maintenance phase. The results demonstrated a significantly greater number of patients in remission in the IN esketamine group compared to patients in the quetiapine group at week 8 (91 of 336 patients [27.1%] vs. 60 of 340 patients [17.6%], respectively, *p* = 0.003).

The long-term efficacy of IN esketamine in terms of response and remission rates and the prevention of relapse was evaluated in three studies: one randomized placebo-controlled trial [36], one randomized active-controlled trial (ESCAPE-TRD) [41], and one open-label study [42]. In ESCAPE-TRD, over 32 weeks of follow-up, the remission rates, response rates, and changes in MADRS scores from baseline were significantly higher in the IN esketamine group than in the quetiapine group. The study also demonstrated that a higher proportion of patients stayed in remission until week 32 without relapsing after having achieved remission at week 8. In the open-label study from Wajs et al. (2020), the improvement in MADRS scores was sustained in responder patients who continued treatment for up to one year of exposure [42]. The percentage of responders and remitters after one year of IN esketamine treatment was 76.5% and 58.2%, respectively. In the randomized placebo-controlled trial by Daly et al. (2019), the time to relapse was significantly longer in patients who received IN esketamine than in patients who received a placebo [36]. Based on hazard ratio (HR) estimation, IN esketamine combined with AD decreased relapse risk by 51% among patients who achieved stable remission and by 70% among patients who achieved stable response compared with patients receiving a placebo combined with AD.

The five observational studies evaluated the effect of IN esketamine in MDD patients with TRD in real-world conditions [43,44,45,46,47].

In the observational study from Brendle et al. (2022), 171 patients received IN esketamine. Significant reductions (*p* < 0.001) in mean Patient Health Questionnaire (PHQ-9) and General Anxiety Disorder (GAD-7) scores from baseline (PHQ-9: mean: 16.7, SD: 5.8; GAD-7: mean: 12.0, SD: 5.8) to last available treatment (PHQ-9: mean: 12.0, SD: 6.4; GAD-7: mean: 8.7, SD: 5.6) were found [45].

The observational study from Martinotti et al. (2022) demonstrated a significant improvement in depressive symptoms with a decrease in MADRS scores from baseline (MADRS: mean: 35 ± 8.53) to 1 month (MADRS: mean: 22.27 ± 9.81, *p* < 0.001) and 3 months (MADRS: mean: 14.69 ± 9.88, *p* < 0.001) follow-up in 117 patients treated with IN esketamine. Furthermore, after 3 months, 64.2% of patients reached a clinical response and 40.6% achieved remission [46].

The real-world cohort study from Samalin et al. 2022 observed that 48% of MDD patients with TRD responded 18 days after the first IN esketamine treatment, and 37% achieved remission after 21 days. Moreover, based on the Kaplan–Meier method, the data suggested that the probability of remission was 31.6% and 60.3% 4 and 8 weeks after the first administration, respectively [47].

The observational study published by Estrade et al. (2023) included 105 MDD patients with TRD. Four weeks after IN esketamine initiation, 52.4% of patients were responders and 38.1% were remitters. Moreover, two classes of trajectories were identified (response and non-response), and the MADRS scores obtained after two IN esketamine administrations predicted the 90-day trajectory of response [43].

In the real-world observational study from Singh et al. (2023), 47 patients received intravenous ketamine and 15 received IN esketamine. The changes in QIDS-SR scores, responses, and remission rates between baseline and the end of the acute phase (approximately 6 weeks) were similar between the groups. Nevertheless, a faster time to remission was observed with intravenous ketamine compared to IN esketamine (HR = 5.0, *p* = 0.02) [44].

#### 3.3.2. Efficacy in MDD Patients with Active Suicidal Ideation

The efficacy of IN esketamine in MDD patients with active suicidal ideation was evaluated in three double-blind, randomized, placebo-controlled trials [31,33,35].

In the ASPIRE I trial, a significant improvement in MADRS scores was observed 24 h after the first dose administration in the IN esketamine group in comparison to the placebo group (mean least-squares (LS) difference [SE]: −3.8 [1.39], *p* = 0.006). Changes in total CGI-SS-r scores at 24 h and 25 days after the first administration reflected an improvement in the severity of suicidality in both groups, but there was no statistical difference [33].

In the ASPIRE II study, the decrease in MADRS scores between baseline and 24 h after the first administration (LS mean difference [SE]: −3.9 [1.39], *p* = 0.006) and day 25 pre-dose (LS mean difference: –3.7, 95% CI: –7.09; –0.38) was significantly greater in the IN esketamine group compared to the placebo group [31]. Patients in both treatment groups experienced a rapid reduction in CGI-SS-r scores, but no statistical difference was observed.

In the third observational study from Canuso et al. (2019), IN esketamine significantly improved depressive symptoms compared to a placebo at different time points after the first drug administration: 4 h (LS mean difference [SE]: −5.3 [2.1], *p* = 0.015), day 1 (LS mean difference [SE]: −7.2 [2.85], *p* = 0.015), day 3 (LS mean difference [SE]: −7.4 [2.92], *p* = 0.015), and day 11 (LS mean difference [SE]: −7.5 [3.29], *p* = 0.029). However, no statistical difference was observed on day 25, the study endpoint (LS mean difference [SE]: −4.5 [3.14], *p* = 0.159) [35]. An improvement in the severity of suicide risk was observed at each time point in both groups but with no statistical significance.

### 3.4. Safety

IN esketamine was generally well tolerated. The most frequently treatment-emergent adverse events (TEAEs) were mild to moderate in terms of severity, generally occurring shortly after the studied drug administration (40 min) and resolved on the day of administration (after 1.5 to 2 h). The most common TEAEs (≥10% of patients) reported during the induction phase were dizziness, dissociation, nausea, headache, somnolence, increased blood pressure, dysgeusia, vertigo, hypoesthesia, vomiting, vision, and paresthesia (Table 3). The 20-item Physician Withdrawal Checklist was used in four RCTs [32,34,37,42]. No withdrawal symptoms were observed 1 or 2 weeks after the cessation of IN esketamine. No studies reported drug abuse or craving during follow-up phases. Adverse events leading to the discontinuation of study drugs occurred in 0.9 to 4.5% of patients receiving a placebo, 11% of patients receiving quetiapine, and 0.9 to 12.2% of patients treated with IN esketamine. Concerning the serious TEAEs, no studies reported any deaths during the double-blind phase. Depression- and suicide-related events were the most frequent serious TEAEs. The percentage of patients having serious TEAEs was not available for all studies. The long-term studies found no new side effects and demonstrated favorable long-term safety and manageable tolerability [36,41,42]. The most frequent TEAEs also were mild to moderate in terms of severity and generally resolved on the day of administration. In the real-world studies, no new safety signals were reported. The side effects observed in these studies were similar to those reported in the RCTs. The most common adverse events were transient increased blood pressure, dissociation, and sedation. These effects were generally transient, appearing on the days of IN esketamine administration and resolving within 2 h after IN esketamine administration [45,46,47].

Evaluations of tolerance to IN ketamine were only based on two small sample size studies, which achieved opposite results. The short-term randomized placebo-controlled trial from Lapidus et al. (2014), using a single fixed dose of 50 mg of IN ketamine, found similar adverse events to those of IN esketamine [23]. The most frequent TEAEs occurred for up to 4 h after IN ketamine administration, and the majority of these symptoms were resolved on the same day. In contrast with these results, the study by Galvez et al. (2018), using a single fixed dose of 100 mg of IN ketamine, found a high rate and intensity of psychotomimetic and general acute side effects [40]. All three participants in the group receiving IN ketamine showed difficulties in the self-administration of sprays due to motor incoordination after only 20 mg of ketamine had been administered. This pilot trial was suspended due to these tolerability problems.

## 4. Discussion

This systematic review of the efficacy and safety of IN ketamine and IN esketamine in MDD patients highlighted the interest in IN formulations as augmentation strategies for oral antidepressants. The efficacy and safety of IN formulations of ketamine and its enantiomer esketamine due to better affinity with NMDA receptors were evaluated in MDD patients with TRD or active suicidal ideation. The evidence of efficacy was stronger for IN esketamine than for IN ketamine in these MDD populations in several randomized placebo-controlled studies and one recent randomized active-controlled study.

To date, there is only one direct comparison study evaluating the efficacy of intravenous ketamine and IN esketamine in a small sample size of MDD patients with TRD [44].

Esketamine is the first and only IN FDA-approved antidepressant for MDD patients with TRD and MDD patients with acute suicidal ideation or behavior. This S-enantiomer of racemic ketamine has demonstrated a significant decrease in depressive symptoms in comparison with placebo groups in several short-term randomized placebo-controlled studies [30,31,33,35,38,39] and has shown superior remission rates at week 8 in comparison to extended-release quetiapine in a randomized active-controlled study of MDD patients with TRD [41].

Long-term studies found a significant increase in the percentage of responders and remitters (up to 76.5% and 58.2%, respectively, [42]) and a reduction in the risk of relapse by 51% among patients who achieved stable remission and 70% among those who achieved stable response in IN esketamine groups in comparison to placebo groups [36]. Three real-world observational studies found similar results, with significant improvements in depressive symptoms at different time points in MDD patients with TRD. One observational study directly compared the efficacy of intravenous ketamine and IN esketamine in a small sample size of MDD patients with TRD. The depressive symptoms evaluated by QIDS-SR showed similar improvements in both groups, independently of the administration route [44].

IN esketamine also demonstrated a significant improvement in depressive symptoms after only 24 h after the first administration in MDD patients with active suicidal ideation in three out of three RCTs [31,33,35]. However, studies found an improvement in suicidal ideation that was not greater than that in the placebo group.

In a very interesting way, data by Fu et al. (2020) and Ionescu et al. (2021) suggest that IN esketamine efficacy is even greater in MDD patients with more severe depressive symptoms and/or who have attempted suicide [31,33].

The efficacy of ketamine has mainly been studied using the intravenous route in MDD patients with TRD and MDD patients with active suicidal ideation [48,49]. Only one RCT with positive results evaluated the efficacy of IN ketamine with a single dose of 50 mg in MDD patients with TRD [23]. The other one included an RCT, which had to be suspended early due to acute problems with tolerability [40]. Another study showed a reduction in active suicidal ideation and improvement in depressive symptoms 4 h post-administration of a single fixed dose of IN ketamine, independently of their psychiatric diagnosis, and was therefore not included in our systematic review [50].

In this systematic review, all patients treated with IN ketamine or IN esketamine received a new oral AD (SSRI or SNRI) or continued an ongoing oral AD (mainly SSRIs or SNRIs) without a combined AD or augmentation agents. The use of IN antidepressant agents as an augmentation approach makes it difficult to distinguish the intrinsic effects of ketamine or esketamine from the positive effects of the combination of ketamine or esketamine with oral ADs. The only available data include a post-hoc analysis of the long-term open-label study that evaluated a subgroup of patients who used IN esketamine as a monotherapy for MDD patients with TRD. The findings showed that depressive symptoms and functioning remained stable during the optimization and maintenance phase [51].

The frequency of IN administrations varied according to the studies. In the study evaluating the efficacy of IN ketamine, MDD patients with TRD only received a single dose and a single drug administration, which means that it is not possible to draw any conclusions regarding the optimal dosage and frequency of use to be considered. Conversely, the RCTs evaluating the efficacy of IN esketamine used the same frequency of administration: two administrations per week during the first 4 weeks of the induction phase, one administration per week during the next 4 weeks of the optimization phase, and one administration per week or every other week during the maintenance phase. The Nijs et al. (2020) post hoc analysis underlines the importance of individualizing IN esketamine treatment frequency to optimize treatment responses in real-world clinical practice [52].

The tolerability of IN esketamine was generally acceptable. The most frequent TEAEs were mild to moderate in terms of severity and they were transient, occurring and being resolved on the day of esketamine administration. The most common side effects reported were dizziness, dissociation, nausea, headache, somnolence, increased blood pressure, dysgeusia, vertigo, hypoesthesia, vomiting, blurred vision, and paresthesia. Evaluations of the safety of IN ketamine found more controversial results. The study from Lapidus et al. (2014) showed similar tolerability with IN esketamine for a single dose of 50 mg of ketamine administered via the IN route [23]. In contrast with these findings, Galvez et al. (2018) found a high frequency of psychotomimetic and general acute side effects as well as patient difficulties with the self-administration of sprays due to motor incoordination as early as the second spray (after 20 mg of ketamine) [40]. The hypothesis suggested by the authors was that the tolerability problems were due to higher plasma concentrations achieved, probably because absorption via the IN mucosa can be too rapid when careful attention is paid to the administration technique. Indeed, the method of administration of an IN drug can also affect how the dose is delivered to the nasal mucosa. In the IN esketamine studies, patients self-administered IN esketamine at the study site under the supervision of a healthcare professional. In the study by Lapidus et al. (2014), IN ketamine administrations were provided over 20 min by an anesthesiologist, whereas, in the study by Galves et al. (2018), patients self-administered IN ketamine under the supervision of a research staff member after extensive training by research staff to ensure the correct insufflation technique and reliable drug administration [23,40].

These findings could also be specific to the IN delivery device used as in these two studies, they were different and used for other compounds (Aptar^®^ IN atomization delivery device, Braeside, VIC, Australia [40] and LMA MADgic^®^ IN mucosal atomization, San Diego, CA, USA, [23]). Furthermore, no PK studies have evaluated the ketamine PK profiles associated with these two IN delivery devices. Conversely, PK and stability studies for market authorization supported the development of an IN delivery device for esketamine [53]. A systematic review of thirteen studies with PK data for healthy subjects and patients with TRD receiving esketamine characterized the PK profiles in plasma following IN administration [28]. After IN administration, the absolute bioavailability was 63%, 54%, and 51% for 28 mg, 56 mg, and 84 mg esketamine doses, respectively. The esketamine PK profile was characterized by fast absorption (0.341 h), high clearance (392 L/h), a mean terminal half-life of 11 h, and a large volume of distribution (752 L). Esketamine exposure increased with the dose from 28 mg to 84 mg. The increase in the concentration maximum (Cmax) and area under the concentration–time–time curve (AUC) was less than dose-proportional between 28 mg and 56 mg or 84 mg, but it was nearly dose-proportional between 56 mg and 84 mg. Following the intranasal administration of esketamine, the time to reach peak plasma concentration (Cmax) was approximately 20 to 40 min post-dose [54]. The systemic bioavailability of esketamine could also be affected by some individual factors, such as environmental pH, mucociliary clearance, or membrane permeability [55]. In addition, the prescribing information recommends starting IN esketamine at 56 mg in adults and making dosage adjustments based on efficacy and tolerability [54].

Lofts et al. have highlighted the benefits of IN treatments, in particular, their efficacy, rapidity of action, safety, and bioavailability for the delivery of therapeutics to the brain [55]. However, the authors also emphasized the need for PK studies of new IN therapies to provide reliable drug administration and dosing parameters. As for esketamine, PK studies of IN ketamine delivery devices should be carried out to provide valuable PK information, and individually titrated dosing rather than a single dose could reduce the risk of side effects.

Furthermore, if the occurrence of (es)ketamine use disorder was not found in the studies, concerns related to the possible sedative or dissociative effects and the potential risk of misuse or abuse reflect that time may be needed for patient education and supervision by a healthcare provider.

No correlation between dose and the occurrence of side effects was found, except in the study by Daly et al. (2018), which showed a correlation between esketamine dose and dizziness [39].

It is worth highlighting that the real-world observational studies did not detect any new safety signals [43,44,45,46,47].

Further research studying the efficacy and tolerability of IN ketamine appears necessary due to the low level of evidence available and the safety concerns found. IN esketamine should also be compared directly with IN ketamine and racemic intravenous ketamine in larger sample populations. Finally, further studies with longer follow-ups are also needed.

This systematic review has some limitations. First, publication bias may have influenced our findings as negative studies on IN ketamine or IN esketamine may not have been published. Second, most of the studies that were included used IN esketamine, while only two used IN ketamine. Furthermore, the main data available for IN ketamine were from Lapidus et al.’s (2014) study, in which participants received a single dose of ketamine, whereas in IN esketamine studies, participants received multiple doses. These limitations highlight the need for further research on IN ketamine (from PK studies to clinical studies). Third, the participants from the RCTs may not be representative of the broader population with TRD because of strict inclusion/exclusion criteria. However, our review also included real-world studies to consider this limit. Finally, it is worth noting that the heterogeneity within the included studies may have influenced our results. For instance, there were different criteria for TRD and mainly short-term RCT studies.

## 5. Conclusions

This systematic review highlighted the interest in IN routes of antidepressant drugs such as ketamine and esketamine for MDD patients with TRD or active suicidal ideation. They provide a rapid onset of antidepressant action within the first hours after administration that can be maintained over time by repeated administration. The safety profile appears to be acceptable for IN esketamine but requires further studies and a more accurate IN delivery device for ketamine.

## Figures and Tables

**Table 3:** Pooled analysis of most frequently reported TEAEs (>10%) in the nine RCTs evaluating the safety of IN esketamine [30,31,32,33,34,35,37,38,39] during the 4 weeks of the induction phase (except for Daly et al., 2018, who evaluated TEAEs during the first 2 weeks [39]).

TEAEs	Placebo n = 782 [30,31,32,33,34,35,37,38,39]	IN Esketamine 28 mg n = 60 [32,39]	IN Esketamine 56 mg n = 176 [32,37,39]	IN Esketamine 84 mg n = 435 [31,32,33,35,37,39]	IN Esketamine Flexible Dose n = 312 [30,34,38]	IN Esktamine Any Doses n = 983 [30,31,32,33,34,35,37,38,39]
**Dizziness**	86 (11.0)	15 (25.0)	58 (33.0)	148 (34.0)	136 (43.6)	357 (36.3)
**Dissociation**	42 (5.4)	14 (23.3)	47 (26.7)	146 (33.6)	114 (36.5)	321 (32.7)
**Nausea**	81 (10.4)	9 (15.0)	42 (23.9)	123 (28.3)	96 (30.8)	270 (27.5)
**Headache**	114 (14.6)	12 (20.0)	31 (17.6)	88 (20.2)	48 (15.4)	179 (18.2)
**Somnolence**	70 (9.0)	10 (16.7)	37 (21.0)	83 (19.1)	35 (11.2)	165 (16.8)
**Increased blood pressure**	40 (5.1)	12 (20.0)	29 (16.5)	57 (13.1)	58 (18.6)	156 (15.9)
**Dysgeusia**	80 (10.2)	2 (3.3)	20 (11.4)	81 (18.6)	46 (14.7)	149 (15.2)
**Vertigo**	9 (1.2)	6 (10.0)	32 (18.2)	51 (11.7)	38 (12.2)	127 (12.9)
**Hypoesthesia**	12 (1.5)	7 (11.7)	22 (12.5)	41 (9.4)	37 (11.9)	107 (10.9)
**Vomiting**	22 (2.8)	1 (1.7)	10 (5.7)	51 (11.7)	39 (12.5)	101 (10.3)
**Blurred vision**	15 (1.9)	0 (0.0)	8 (4.5)	38 (8.7)	37 (11.9)	83 (8.4)
**Paresthesia**	14 (1.8)	0 (0.0)	19 (10.8)	40 (9.2)	17 (5.4)	76 (7.7)

All data are n (%).

## Data Availability

Not applicable.
